# Subjective social status and functional and mobility impairments among older adults: life satisfaction and depression as mediators and moderators

**DOI:** 10.1186/s12877-023-04380-5

**Published:** 2023-10-23

**Authors:** Manacy Pai, T. Muhammad

**Affiliations:** 1 Department of Sociology and Criminology, Kent State University, Kent, OH 44242 USA; 2 Department of Family & Generations, International Institute for Population Sciences, Mumbai, 400088 India

**Keywords:** Subjective social status, Functional and mobility impairment, Older adults

## Abstract

**Background:**

While functional and mobility impairments (FMIs) have garnered the attention of health researchers in low and middle-income countries (LMICs), including India, research has yet to explore whether and to what extent the perception of one’s social status is associated with FMIs. We fill this gap in the literature by examining (1) the association between subjective social status (SSS) and FMIs among older adults in India and (2) whether this association between SSS and FMIs is mediated and moderated by life satisfaction and depression.

**Methods:**

Data come from the 2017-18 wave 1 of the Longitudinal Aging Study in India (LASI) with a sample of 31,464 older adults aged 60 years and above. FMIs were assessed using established scales on impairments in activities of daily living (ADLs), instrumental activities of daily living (IADLs), and mobility. SSS was assessed using the Macarthur scale. Life satisfaction was measured using responses to five statements gauging respondent’s overall satisfaction with life. Depression was calculated using the shortened version of the Composite International Diagnostic Interview (CIDI-SF). Multivariable regression was employed to examine the association between variables, and the interaction terms and Karlson-Holm-Breen (KHB) method were used separately to test the mediation and moderation effects.

**Results:**

39.11% of the sample had a low SSS, 8.26% were depressed, and 32.07% reported low life satisfaction. A total of 8.74%, 10.91%, and 8.45% of the study population reported at least one impairment in ADL, IADL, and mobility, respectively. Older adults in the higher SSS group were less likely to have ADL impairment (beta: -0.017, CI: -0.030, -0.0032) and mobility impairment (beta: -0.044, CI: -0.076, -0.013). Depression moderated the association between SSS and mobility impairment (p-value: 0.025), and life satisfaction moderated the association between SSS and ADL impairments (p-value: 0.041) and SSS and IADL impairments (p-value: 0.037). Depression mediated 20.28%, 31.88%, and 18.39% of the associations of SSS with ADL, IADL, and mobility impairments, respectively. Similarly, life satisfaction mediated 23.24%, 52.69%, and 27.22% of the associations of SSS with ADL, IADL, and mobility impairments.

**Conclusions:**

That SSS is associated with FMIs among older Indians, even after considering their objective socioeconomic status (SES), suggests that the use of SSS is relevant to the study of health inequalities in India. The finding that life satisfaction and depression mediate and moderate this association is crucial in pinpointing those older Indians at risk of the functional and mobility-related repercussions of lower SSS.

## Background

Functional and mobility impairments (FMIs) are important health-related challenges in later life [1, 2]. FMIs, which are assessed in many ways, driven by the conceptual models of disablement, indicate one’s ability to function independently [3–8]. Given that they compromise independence, reduce social engagement [1, 9], and elevate the risk for falls [1], restricted access to medical services [10, 11], and premature mortality [12–14], FMIs constitute a major challenge to the health care systems worldwide [14, 15]. While high-income nations have had the time to acclimate to such challenges, they remain particularly daunting for low and middle-income countries (LMICs) like India that are being pummeled by the simultaneous increases in aging adults and chronic diseases [16–18] – both likely to increase the prevalence of FMIs.

A recent study reported that 44% of older adults in India live with functional impairments, as measured by impairments in one or more activities of daily living (ADLs), which include essential competencies for survival, such as personal hygiene, continence, dressing, bathing, ambulating or transferring; and instrumental activities of daily living (IADLs), which are essential for independent living and include managing communication with others, finances, medications, food preparation, and housekeeping [19–22]. Another study found 57% and 73% of older Indians to have high IADL and bodily impairment scores [23]. ADL and IADL assessments are critical in determining if older adults can “age in place” or require formal care assistance, including skilled nursing. For instance, a reduced ADL capacity to move about may raise the risk of falling [8, 20, 24]. Similarly, impaired ability to carry out IADLs, like managing medications, adversely impacts health in older adults [25, 26]. Given this, identifying factors that render older adults susceptible to FMIs is critical to crafting interventions that protect them against physical decline. One factor, which remains overlooked within the Indian context, is subjective social status (SSS).

SSS is an individual’s perceived social status compared to others in their community [27]. Individuals with higher SSS report better health than their lower-SSS peers [28–30]. SSS often predicts health above and beyond the objective indicators of socioeconomic status (SES), including education, occupation, income, wealth, and even caste [29–32]. This may be because SSS encapsulates the psychosocial aspects associated with SES, such as power, prestige, ties to the mainstream, society, perceived fairness, and status internalization [30, 33–35]. Such less obvious aspects related to an individual’s sense of self and identity are not necessarily captured by objective markers of SES that are restricted to static positions in one’s life [36–38]. For instance, occupation and income may not accurately reflect later life SES, given that many older adults, especially in LMICs like India, may be retired or financially dependent on their adult children [37, 38]. Likewise, for older Indian women with constrained opportunities for higher education and paid work [39], education and occupational ranking may not accurately represent their SES. As such, it is critical to gauge the association between SSS and FMIs in resource restrained countries.

Low SSS, which often is linked to perceived unfairness, can cause “psychological pain” and set into motion adverse physiological stress reactions (e.g., hypervigilance, increased respiration and heart rate, elevated blood pressure, sleep disturbances, cortisol secretions, and chronic inflammation) [35, 40, 41], and these amplified physiological responses over time can debilitate physical function and mobility. Low SSS may also engender a perceived lack of power to change one’s life circumstances, which could prompt risky health behaviors [42, 43] and prevent health-promoting actions [42], which can shape functional health. The study of stress, no doubt, identifies SSS as a powerful social stressor affecting later life functionality [31, 44, 45].

That said, the stress process framework highlights the centrality of individual characteristics as potential mediators and moderators of the association between stressors and distress [46]. Two such characteristics are life satisfaction and depression. While high life satisfaction, which encompasses feelings of autonomy, openness, optimism, and adaptation [47–49], is a resource for maintaining functional health and mobility, low life satisfaction, which is associated with higher levels of physiological stress, psychosocial, and behavioral problems [50], may increase the risk for FMIs. While high levels of depression result in internalizing behaviors of self-blame, denial, rumination, withdrawal, and self-neglect [51], which can increase the risk of FMIs, individuals with lower depression may remain at relatively minimal risk of FMIs. Both high life satisfaction [49, 52] and less depression [53] involve preemptive coping, which increases stress resistance that may be tied to lower SSS. Older adults who are more satisfied with life may adapt better to life circumstances and problem-solve challenges attached to material hardships underlying SSS. Similarly, those less depressed may cognitively reinterpret challenges [54, 55] related to low SES as more manageable, concentrating on those aspects of their SES they believe to be less intractable.

**Fig. 1:**
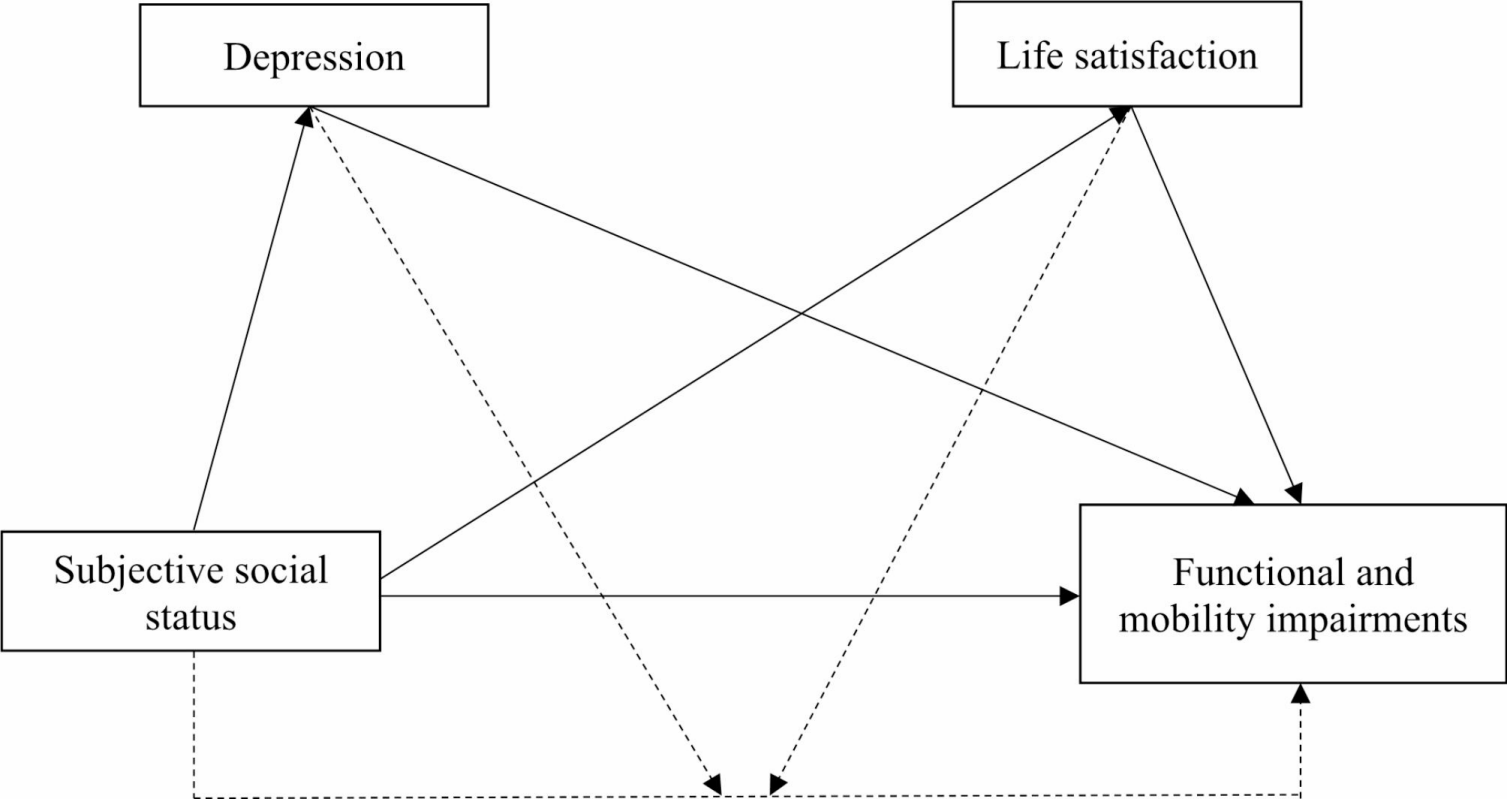
Conceptual framework of the study *Notes: Dashed arrows indicate the interaction effects (depression and life satisfaction affecting the association between SSS and FMI) whereas, continuous lines indicate the main effects, either direct associations (SSS and FMI) or meditational pathways (i.e., depression and life satisfaction as mediators of the association between SSS and FMI)*

In sum, the present study hypothesizes that older adults with lower SSS are more likely to report more FMIs. We also hypothesize that both life satisfaction and depression mediate this association. Statistically, this implies that the SSS coefficients should attenuate once we adjust for life satisfaction and depression. Lastly, we posit that life satisfaction and depression also moderate the association between SSS and FMIs, such that this association is less pronounced among older adults with higher life satisfaction and lower depression than their peers who report lower life satisfaction and higher levels of depression. Figure 1*summarizes the concept of our study*.

## Methods

### Study participants

The present study utilizes the individual-level data from the first wave of the Longitudinal Aging Study in India (LASI) conducted during 2017-18. The LASI is a country-representative longitudinal survey of more than 72,000 adults aged 45 years and over across all states and union territories of the country that provides vital information on the social, physical, psychological, and cognitive health of the Indian aging population. The LASI survey was conducted through a partnership of the International Institute for Population Sciences (IIPS), Harvard T. H. Chan School of Public Health (HSPH), and the University of Southern California (USC). In the LASI wave 1, the sample selection is based on a multistage stratified cluster sample design, including a three-stage sampling design in rural areas and a four-stage sampling design in urban areas. The details of the sampling design, survey instruments, and data collection procedures are provided elsewhere [16, 56]. The present study is conducted on the eligible respondents aged 60 years and above. Thus, the total sample size for the present study was 31,464 (15,098 men and 16,366 women) older adults aged 60 years and above.

### Measures

The description of the study variables and our rationale for the selected covariates are provided.

below in Table 1.

**Table 1:** Description of the measures included in the study

Variables	Description of the variable and the categories	
Outcome variables		
ADL impairment	ADL is a term that refers to normal daily self-care activities (walking across a room, dressing, bathing, eating, getting in and out of bed, and toileting). Cronbach alpha value that assesses the internal consistency of the ADL scale was 0.87. Impairment in ADL is measured as a difficulty in any of these activities that lasts for a minimum three months’ period. Given that ADL is a barometer for an individual’s functional status [22], prolonged impairment in ADLs may mean increased dependence on others and/or mechanical devices.	
IADL impairment	Impairment in IADL was measured as a difficulty in any of the following activities that lasts for three months or more; preparing a hot meal (cooking and serving), shopping for groceries, making a telephone call, taking medications, doing work around the house or garden, managing money (such as paying bills and keeping track of expenses), and getting around or finding an address in unfamiliar places (Cronbach alpha value for the IADL scale was 0.88).	
Mobility impairment	Mobility impairment was assessed using nine items that include difficulty in walking 100 yards, sitting for 2 h or more, climbing one flight of stairs without resting, getting up from a chair after sitting for long period, reaching or extending arms above shoulder level (either arm), stooping, kneeling or crouching, pulling or pushing large objects, lifting or carrying weights over 5 kilos, like a heavy bag of groceries and picking up a coin from a table (Cronbach alpha value was 0.87). In this study, impairment scores for ADL, IADL and mobility were measured using a numerical continuous scale. ADL impairment scale ranges 0–6, IADL impairment scale ranges 0–7 and mobility impairment scale ranges 0–9, with 0 indicating no impairment and the highest scores on each reflecting the most severe impairment on each measure.	
Explanatory variables		
Subjective social status	SSS in this study was assessed using the Macarthur scale [57], with a ladder technique, and the question used to assess the variable was, “Think of the ladder with 10 stairs as representing where people stand in our society.” At the top of the ladder are the people who are best off – those who have the most money, the most education, and the best jobs. At the bottom are the people who are the worst off – those who have the least money, the least education, and the worst jobs or no jobs. “The higher up you are on this ladder, the closer you are to the people at the very top, and the lower you are, the closer you are to the people at the very bottom of your society” [56]. The scale is used to measure the subjective SES across different populations in India and other countries [58–60]. The respondent was instructed to “Please indicate the number given on the rung on the ladder where you would place yourself” (Interviewer needs to fill the number in the box given in the side of the ladder). A score of 1–10 was generated as per the number of rungs marked by the respondents and was used as a continuous measure in the analysis. A score of 8–10 was considered “high”, 4–7 was considered “middle” and 1–3 was considered “low” in the current analysis.	
Mediator/Moderator variables		
Depression	Depression was calculated using the Short Form Composite International Diagnostic Interview (CIDI-SF) with ten items, including three screening questions and seven symptoms (Cronbach alpha value was 0.69). A scale that ranges from 0 to 10 was constructed where higher score reflects higher level of depression. We employed five as a cut-off point for depression that leads to a 0.89 probability of CIDI caseness of major depression [61, 62]. The CIDI-SF scale estimates a probable psychiatric diagnosis of major depression and has been validated in field settings and widely used in population-based health surveys [63–66].	
Life satisfaction	Life satisfaction was assessed using the following questions: (a) In most ways, my life is close to ideal; (b) The conditions of my life are excellent; (c) I am satisfied with my life; (d) So far, I have got the important things I want in life; (e) If I could live my life again, I would change almost nothing These 5 items are added to create an index of life satisfaction. The Cronbach alpha for the reliability test of this index variable was 0.90. The responses were recorded using a 7-point Likert scale (1 = strongly disagree, 7 = strongly agree), where higher number reflects higher levels of life satisfaction (range 5–35). It was further categorised into low (5–20), medium (20–25) and high (25–35).	
Covariates		
Age		In years
Gender		Male; female
Education		No formal education, primary, secondary, higher
Marital status		Currently married, widowed, divorced/separated/never married
Living arrangements		Living alone, with spouse and others
Work status		Never worked, currently not working, currently working, and retired. Work status was identified from the survey questions “Have you ever worked for at least 3 months during your lifetime?”, “Are you currently working?” and “Did you ever officially retire from the organized sector of employment?”
Monthly per-capita consumption expenditure (MPCE) quintile		Based on recommendations for “better” indicators of SES in LMICs [67], older adults’ SES was assessed using the monthly per-capita consumption expenditure (MPCE) quintile. Sets of 11 and 29 questions on the expenditures on food and non-food items, respectively, are used to canvass the sample households. Food expenditure was collected based on a reference period of seven days, while the non-food expenditure was collected using reference periods of 30 days and 365 days [68]. Food and non-food expenditures have been standardized to the 30-day reference period. The variable is divided into five quintiles i.e., from poorest to richest.
Multimorbidity		Given the association of chronic conditions with functional ability [69], we considered having multimorbidity as a confounder in this study. It refers to the coexistence of two or more chronic diseases, which include hypertension, diabetes, cancer, chronic heart diseases, stroke, chronic lung disease, bone/joint disease, and neurological/psychiatric disease. These diseases were assessed using the survey question, ‘Has any health professional ever diagnosed you with the following chronic conditions or diseases?’
Community involvement		As documented in previous studies, social support and networks have a positive influence on older adults’ functional ability [70]. Therefore, we considered community involvement as a confounder in our analysis which was assessed using the survey question, “Are you member of any social organizations, religious groups, clubs or societies?” and coded as yes or no.
Religion		Hindu; Muslim; Christian; Others
Caste		Scheduled castes (SC); scheduled tribe (ST); other backward classes (OBC); others. The SC refers to the population that is socially segregated and financially/economically weak by their low status as per the caste hierarchy. Similarly, the ST refers to the indigenous populations who are considered among the most disadvantaged and discriminated socio-economic groups in the country. The OBC is the group of people who are identified as “socioeconomically and educationally backwards.” The ‘other’ caste category is identified as having higher social status, mostly belong to upper caste categories [71].
Place of residence		Prior research has found that the prevalence of both ADLs and IADLs is significantly higher among rural adults compared to their urban dwelling counterparts [72]. It also is more generally found that older adults in rural regions of India are more socioeconomically distressed and endure more health care challenges, rendering them more vulnerable to ADLs and IADLs relative to their peers in urban settings [73–75]. Given this, we consider place of residence -- coded as urban versus rural -- in our analysis.
Regions		Recent research has found that older adults in southern India report more poor self-rated health [76] and functional difficulty [77] than their older peers in Northern and other regions of the country. Therefore, our analysis does account for regional variations in the association between SSS and FMI. The regions of the country are coded as North, Central, East, Northeast, West, and South.

### Statistical analysis

We presented the results from descriptive statistics and bivariate analysis (cross-tabulation) and p-values from Chi-Square tests, indicating the statistical significance of the differences in outcome variables across the selected background variables. Multivariable logistic regression analysis was performed to determine the association between the outcome variables (ADL/IADL/mobility impairments) and SSS, depression, and life satisfaction. Further, interaction analyses of SSS, depression and life satisfaction on outcome variables were conducted, and margin plots were presented. As a post-hoc analysis, interaction terms and the Karlson–Holm–Breen (KHB) method were used separately to examine the effect modification and percent mediation by depression and life satisfaction in the association between SSS and outcome variables. The estimates were presented as adjusted beta coefficients with a 95% confidence interval. Statistical models were adjusted for the selected predictor variables, including age, gender, schooling, marital status, living arrangement, work status, multimorbidity, community involvement, household consumption quintiles, religion, caste, place of residence, and region of the country. Individual weights were applied during the analysis to account for the cluster sampling and to provide the population-level estimates. The statistical analysis was performed using Stata 15.1.

## Results

Table 2 presents the sample characteristics. A proportion of 39.1% of the sample had a low SSS, whereas only 7.3% had a high SSS in this study. 8.3% of the sample was depressed, and 32.1% reported low life satisfaction. More than 10% of the sample was aged 80 years or older and a significant proportion (56.4%) had no formal education. 36% of the sample was widowed, and 26.5% reported never having worked more than three months during their lifetime.

**Table 2:** Sample distribution by background variables and SSS

Background variables	Sample	SSS		
		Low	Medium	High
	n (w %)	n (w %)	n (w %)	n (w %)
SSS				
Low (1–3)	10,661 (39.11)			
Medium (4–7)	17,233 (53.60)			
High (8–10)	2359 (7.29)			
Depression				
No	28,110 (91.35)	9599 (88.13)	16,243 (93.16)	2268 (95.27)
Yes	2132 (8.26)	1060 (11.87)	983 (6.84)	89 (4.73)
Life satisfaction				
Low (5–20)	9216 (32.07)	4772 (44.11)	4137 (25.95)	307 (12.45)
Middle (21–25)	7197 (22.37)	2423 (22.06)	4380 (23.88)	394 (12.97)
High (26–35)	13,794 (45.56)	3449 (33.83)	8687 (50.17)	1658 (74.58)
Age (In years)				
60–69 years	18,473 (59.5)	6462 (58.85)	10,559 (59.73)	1452 (61.28)
70–79 years	8718 (29.8)	3091 (30.14)	4935 (29.95)	692 (26.82)
80 + years	3062 (10.71)	1108 (11.01)	1739 (10.32)	215 (11.91)
Level of education				
No formal education	16,135 (56.38)	7281 (70.25)	8118 (50.05)	736 (28.45)
Primary	5652 (17.68)	1903 (16.59)	3393 (18.95)	356 (14.19)
Secondary	5918 (18.09)	1251 (11.12)	3961 (21.29)	706 (31.99)
Higher	2548 (7.85)	226 (2.04)	1761 (9.71)	561 (25.38)
Gender				
Men	14,513 (47.24)	4733 (44.35)	8484 (48.17)	1296 (55.9)
Women	15,740 (52.76)	5928 (55.65)	8749 (51.83)	1063 (44.1)
Marital status				
Currently married	19,312 (61.85)	6301 (58.09)	11,309 (63.22)	1702 (72.03)
Widowed	10,161 (36.01)	4033 (39.37)	5521 (34.92)	607 (26.01)
Divorced/ separated/ never married	780 (2.13)	327 (2.54)	403 (1.86)	50 (1.96)
Living arrangement				
Alone	1557 (5.7)	831 (8.31)	652 (4.11)	74 (3.39)
With spouse	5906 (19.82)	2222 (21.57)	3217 (18.97)	467 (16.72)
Others	22,790 (74.47)	7608 (70.12)	13,364 (76.91)	1818 (79.89)
Work status				
Never worked	8451 (26.51)	2597 (22.66)	5106 (28.71)	748 (31)
Currently not working	10,421 (35.56)	4165 (39.8)	5629 (33.37)	627 (28.87)
Currently working	8779 (30.58)	3586 (34.6)	4673 (28.92)	520 (21.25)
Retired	2602 (7.35)	313 (2.94)	1825 (9)	464 (18.88)
Multimorbidity				
No	22,774 (76.16)	8436 (79.94)	12,659 (74.1)	1679 (71.05)
Yes	7462 (23.84)	2218 (20.06)	4565 (25.9)	679 (28.95)
Community involvement				
No	28,127 (95.22)	10,084 (96.7)	15,886 (94.32)	2157 (93.83)
Yes	2100 (4.78)	567 (3.3)	1333 (5.68)	200 (6.17)
Household consumption quintiles				
Poorest	6185 (21.79)	2970 (27.38)	2897 (19.01)	318 (12.16)
Poorer	6221 (21.76)	2521 (24.25)	3340 (20.77)	360 (15.63)
Middle	6188 (20.73)	2104 (20.13)	3647 (20.81)	437 (23.42)
Rich	5948 (19.2)	1813 (17.53)	3649 (20.25)	486 (20.43)
Richest	5711 (16.53)	1253 (10.72)	3700 (19.15)	758 (28.35)
Religion				
Hindu	22,208 (82.69)	8059 (81.56)	12,374 (82.98)	1775 (86.63)
Muslim	3576 (10.74)	1359 (11.96)	1996 (10.32)	221 (7.37)
Christian	2976 (2.87)	799 (3.09)	1920 (2.65)	257 (3.35)
Others	1493 (3.69)	444 (3.39)	943 (4.05)	106 (2.65)
Caste				
SC	4941 (19)	2377 (24.85)	2359 (16.03)	205 (9.44)
ST	4935 (8.07)	1911 (11.37)	2714 (6.17)	310 (4.36)
OBC	11,454 (45.14)	4087 (43.25)	6490 (46.39)	877 (46.2)
General	8923 (27.78)	2286 (20.53)	5670 (31.4)	967 (40)
Place of residence				
Urban	10,294 (28.93)	2537 (19.15)	6617 (33.17)	1140 (50.2)
Rural	19,959 (71.07)	8124 (80.85)	10,616 (66.83)	1219 (49.8)
Region				
North	5644 (12.82)	1869 (11.9)	3289 (13.35)	486 (13.88)
Central	4085 (20.97)	1987 (26.67)	1851 (17.3)	247 (17.43)
East	5591 (23.9)	2431 (26.88)	2777 (22.31)	383 (19.65)
Northeast	3573 (2.99)	859 (2.1)	2397 (3.54)	317 (3.72)
South	7237 (22.01)	2323 (18.92)	4372 (23.96)	542 (24.22)
West	4123 (17.3)	1192 (13.54)	2547 (19.53)	384 (21.11)
Total	30,253 (100)			

n: un-weighted sample counts; w %: weighted percentages, to account for complex survey design and to provide population estimates; SC: Scheduled Caste; Scheduled Tribe; OBC: Other backward class

Table 3 presents the prevalence of ADL/IADL and mobility impairments among the sample population. A total of 8.7%, 10.9%, and 8.5% of the study population reported at least one impairment in ADL, IADL, and mobility, respectively, whereas 8%, 25.5%, and 56.2% of older adults reported more than two impairments in ADL, IADL and mobility, respectively. Those with a high SSS (4.2%, 18.2%, and 49.6%) or high life satisfaction (5.6%, 20.5%, and 52%) had a lower prevalence of ADL, IADL and mobility impairments. Alternatively, those who were depressed had a higher prevalence of ADL, IADL, and mobility impairments (18.7%, 43%, and 74.4%).

**Table 3:** Prevalence estimates of ADL/IADL/mobility impairments among older adults by background variables

	ADL impairment				IADL impairment				Mobility impairment			
Background variables	Single	Two	Two plus	p-value	Single	Two	Two plus	p-value	Single	Two	Two plus	p-value
SSS				< 0.001				< 0.001				< 0.001
Low (1–3)	8.71	5.25	8.56		11.48	8.69	28.84		8.16	8.32	59.6	
Medium (4–7)	8.79	4.49	6.52		11.13	7.57	22.46		8.66	9.74	54.09	
High (8–10)	8.32	3.39	4.16		8.92	6.45	18.22		9.5	8.66	49.6	
Depression				< 0.001				< 0.001				< 0.001
No	8.5	4.41	6.23		10.97	7.82	23.18		8.76	9.35	54.32	
Yes	11.84	7.74	18.66		12.18	8.39	43.04		5.58	6.13	74.42	
Life satisfaction				< 0.001				< 0.001				< 0.001
Low (5–20)	8.1	5.3	8.88		10.9	8.65	29.2		7.39	8.12	59.91	
Medium (21–25)	9.08	4.74	7.54		11.8	7.98	26.1		8.42	9.18	57.73	
High (26–35)	8.97	4.14	5.62		10.79	7.32	20.54		9.37	9.78	51.95	
Age (In years)				< 0.001				< 0.001				< 0.001
60–69 years	7.76	3.61	4.57		11.31	7.57	17.95		9.76	10.11	48.09	
70–79 years	10.16	5.88	9.83		11.42	8.61	30.94		7.34	8.07	64.82	
80 + years	10.47	7.21	21.98		7.28	6.92	53.51		4.06	5.52	78.71	
Level of education				< 0.001				< 0.001				< 0.001
No formal education	8.93	5.13	9.35		10.99	8.73	34.09		7.42	8.24	60.86	
Primary	9.83	4.83	7.46		11.86	8.71	20.6		8.77	9.3	56.81	
Secondary	7.69	3.87	5.96		10.51	5.89	13.6		9.74	10.47	49.26	
Higher	7.55	3.07	4.75		9.2	4.29	9.16		11.31	10.16	41.2	
Gender				< 0.001				< 0.001				< 0.001
Men	7.81	3.6	6.71		10.87	6.45	17.8		10.17	9.62	47.55	
Women	9.61	5.62	9.11		10.94	9.05	32.65		6.86	8.48	64.21	
Marital status				< 0.001				< 0.001				< 0.001
Currently married	8.99	4.92	6.06		11.61	7.55	21.4		9.07	9.38	52.33	
Widowed	10.11	7.22	10.14		10.41	8.19	40.45		6.94	7.97	68.83	
Divorced/ separated/ never married	10.76	2.3	9.67		10.48	10.99	29.6		9.57	8.46	54.11	
Living arrangement				< 0.001				< 0.001				< 0.001
Alone	10.01	6.24	8.65		11.52	10.59	30.71		6.55	7.97	64.15	
With spouse	7.91	4.14	6.37		11.41	8.38	18.81		9.05	8.87	52.28	
Others	8.87	4.68	8.33		10.74	7.46	26.92		8.42	9.14	56.71	
Work status				< 0.001				< 0.001				< 0.001
Never worked	9.02	6.27	8.9		9.77	7.64	36.22		6.18	7.91	66.89	
Currently not working	10.64	7.59	11.12		10.14	8.51	37.42		6.16	8.03	67.45	
Currently working	8.11	3.19	2.38		13.48	8.08	14.16		12.19	10.6	42.39	
Retired	10.52	4.79	7.66		11.37	4.52	16.31		10.29	8.96	49.36	
Multimorbidity				< 0.001				< 0.001				< 0.001
No	9.22	4.83	5.95		11.23	7.61	25.74		8.89	9.53	53.75	
Yes	10.12	8.45	12.9		10.86	8.65	37.06		6.46	6.63	72.93	
Community involvement												
No	9.52	5.82	7.81		11.11	7.91	29.14		8.21	8.75	58.87	
Yes	7.57	3.17	3.63		12.01	6.73	14.72		10.28	10.8	47.32	
Household consumption quintiles				0.149				0.041				0.096
Poorest	8.65	4.89	8.79		9.77	7.71	29.13		7.56	8.93	56.31	
Poorer	8.53	4.59	7.57		11.26	8.01	25.98		8.59	9.04	55.86	
Middle	9.11	4.71	7.72		10.75	7.95	24.73		9.43	8.71	56.08	
Rich	8.91	4.71	7.35		11.06	7.76	24.78		8.5	9.18	56.98	
Richest	8.51	4.35	8.39		11.78	7.57	22.75		8.15	9.3	55.9	
Religion				< 0.001				< 0.001				< 0.001
Hindu	9.1	4.89	7.93		10.89	7.96	26.11		8.75	8.91	56.47	
Muslim	9.15	4.6	9.72		10.12	7.7	29.94		7.24	9.02	61.24	
Christian	6.66	3.03	7.04		8.84	7.28	18.99		6.47	9.02	51.18	
Others	6.63	4.55	6.05		17.28	6.84	19.56		10.92	10.79	50.81	
Caste				< 0.001				< 0.001				< 0.001
SC	8.43	5.07	8.27		12.17	8.08	27.44		9.09	9.54	56.65	
ST	7.26	3.1	6.75		9.33	7.2	22.51		7.26	9.1	49.57	
OBC	9.32	4.51	7.78		10.78	8.53	27.2		9.19	8.76	56.48	
General	9.01	5.47	8.7		11.26	7.05	24.01		7.8	9.05	59.38	
Place of residence				0.355				< 0.001				< 0.001
Urban	8.94	4.51	8.24		10.77	6.73	20.71		8.65	9.66	54.37	
Rural	8.64	4.73	7.82		10.98	8.36	28.02		8.34	8.7	57.18	
Region				< 0.001				< 0.001				< 0.001
North	6.54	3.25	6.72		11.83	6.61	23.64		8.6	8.6	52.23	
Central	8.29	3.43	7.85		9.3	7.45	26.22		7.94	7.57	54.75	
East	10.66	6.6	8.6		10.99	8.1	28.74		7.64	9.18	63.29	
Northeast	5.98	2.48	4.97		8.01	6.65	20.49		7	9.56	49.48	
South	7.96	4.34	8.64		11.32	9.27	28.87		9.54	9.13	56.64	
West	13.41	7.64	10.34		12.98	7.8	21.58		9.17	10.22	58.79	
Total	8.74	4.66	7.96		10.91	7.8	25.53		8.45	9.03	56.22	

P-values are based on Chi-Square test; SC: Scheduled Caste; Scheduled Tribe; OBC: Other backward class

Table 4 presents the estimates from the multivariable analysis. Older adults in the higher SSS group were less likely to have ADL impairment (beta: -0.017, CI: -0.030, -0.0032) and mobility impairment (beta: -0.044, CI: -0.076, -0.013). Older adults who were depressed were more likely to have ADL (beta: 0.071, CI: 0.056, 0.086), IADL (beta: 0.12, CI: 0.096, 0.14), and mobility impairments (beta: 0.14, CI: 0.11, 0.16). Alternatively, those who report a higher level of life satisfaction were less likely to have any ADL (beta: -0.012, CI: -0.015, -0.0079), IADL (beta: -0.014, CI: -0.022, -0.0056) and mobility impairments (beta: -0.019, CI: -0.026, -0.011) in this study.

**Table 4:** Multivariable OLS regression estimates of ADL/IADL/mobility impairments among older adults by background variables

Variables	ADL impairment		IADL impairment		Mobility impairment		
	Beta	95% Confidence interval	Beta	95% Confidence interval	Beta	95% Confidence interval	
Ladder SES (1–10)	-0.017*	(-0.030 - -0.0032)	-0.0048	(-0.038–0.029)	-0.044**	(-0.076 - -0.013)	
Depression	0.071***	(0.056–0.086)	0.12***	(0.096–0.14)	0.14***	(0.11–0.16)	
Life satisfaction	-0.012***	(-0.015 - -0.0079)	-0.014**	(-0.022 - -0.0056)	-0.019***	(-0.026 - -0.011)	
Age (In years)	0.033***	(0.029–0.038)	0.069***	(0.062–0.077)	0.081***	(0.072–0.089)	
Schooling (In years)	-0.013***	(-0.019 - -0.0065)	-0.053***	(-0.067 - -0.040)	-0.036***	(-0.050 - -0.022)	
Gender							
Men	Ref.		Ref.		Ref.		
Women	0.024	(-0.037–0.085)	0.47***	(0.36–0.58)	0.58***	(0.44–0.72)	
Marital status							
Currently married	Ref.		Ref.		Ref.		
Widowed	0.0079	(-0.056–0.072)	0.23***	(0.095–0.36)	0.16*	(0.018–0.31)	
Divorced/ separated/ never married	0.084	(-0.071–0.24)	0.25*	(0.0072–0.50)	-0.096	(-0.40–0.21)	
Living arrangement							
Alone	Ref.		Ref.		Ref.		
With spouse	0.10	(-0.020–0.23)	0.18	(-0.043–0.40)	0.037	(-0.22–0.30)	
Others	0.082	(-0.026–0.19)	0.32**	(0.12–0.53)	0.021	(-0.20–0.24)	
Work status							
Never worked	Ref.		Ref.		Ref.		
Currently not working	0.028	(-0.046–0.10)	0.17*	(0.016–0.32)	0.25**	(0.099–0.41)	
Currently working	-0.26***	(-0.33 - -0.20)	-0.49***	(-0.63 - -0.34)	-0.81***	(-0.98 - -0.65)	
Retired	0.020	(-0.098–0.14)	-0.054	(-0.28–0.17)	-0.18	(-0.43–0.069)	
Multimorbidity							
No	Ref.		Ref.		Ref.		
Yes	0.35***	(0.28–0.41)	0.52***	(0.39–0.64)	1.10***	(0.97–1.22)	
Community involvement							
No	Ref.		Ref.		Ref.		
Yes	-0.11**	(-0.18 - -0.041)	-0.31***	(-0.43 - -0.19)	-0.34***	(-0.54 - -0.14)	
Household consumption quintiles							
Poorest	Ref.		Ref.		Ref.		
Poorer	-0.060	(-0.13–0.0063)	-0.096	(-0.22–0.026)	-0.030	(-0.17–0.11)	
Middle	-0.033	(-0.11–0.041)	-0.19**	(-0.32 - -0.062)	-0.028	(-0.19–0.13)	
Rich	-0.089*	(-0.16 - -0.015)	-0.13	(-0.30–0.033)	-0.050	(-0.21–0.11)	
Richest	-0.015	(-0.11–0.081)	-0.18*	(-0.33 - -0.031)	-0.0054	(-0.20–0.19)	
Religion							
Hindu	Ref.		Ref.		Ref.		
Muslim	0.026	(-0.044–0.095)	-0.0081	(-0.13–0.11)	0.19*	(0.022–0.35)	
Christian	0.14*	(0.016–0.27)	-0.32**	(-0.52 - -0.12)	-0.14	(-0.39–0.10)	
Others	0.029	(-0.10–0.16)	0.017	(-0.19–0.22)	-0.053	(-0.27–0.17)	
Caste							
SC	Ref.		Ref.		Ref.		
ST	-0.090	(-0.18–0.00048)	-0.065	(-0.22–0.091)	-0.31**	(-0.52 - -0.11)	
OBC	-0.081*	(-0.15 - -0.010)	0.012	(-0.11–0.13)	-0.13	(-0.27–0.0054)	
General	-0.017	(-0.093–0.060)	-0.053	(-0.17–0.066)	0.083	(-0.061–0.23)	
Place of residence							
Urban	Ref.		Ref.		Ref.		
Rural	0.085**	(0.029–0.14)	0.43***	(0.33–0.53)	0.30***	(0.17–0.42)	
Region							
North	Ref.		Ref.		Ref.		
Central	0.14***	(0.071–0.21)	0.13*	(0.015–0.25)	0.51***	(0.35–0.67)	
East	0.30***	(0.23–0.37)	0.43***	(0.32–0.54)	0.83***	(0.70–0.97)	
Northeast	0.071	(-0.00041–0.14)	0.25***	(0.11–0.39)	0.67***	(0.49–0.84)	
South	0.14***	(0.072–0.21)	0.64***	(0.48–0.80)	0.61***	(0.45–0.77)	
West	0.51***	(0.43–0.59)	0.28***	(0.16–0.40)	0.96***	(0.80–1.12)	
Observations	30,150		30,111		30,150		
R-squared	0.124		0.215		0.194		

*<0.05; **<0.01; ***<0.001; Beta coefficients are adjusted for all the selected covariates; SC: Scheduled Caste; Scheduled Tribe; OBC: Other backward class

**Fig. 2:**
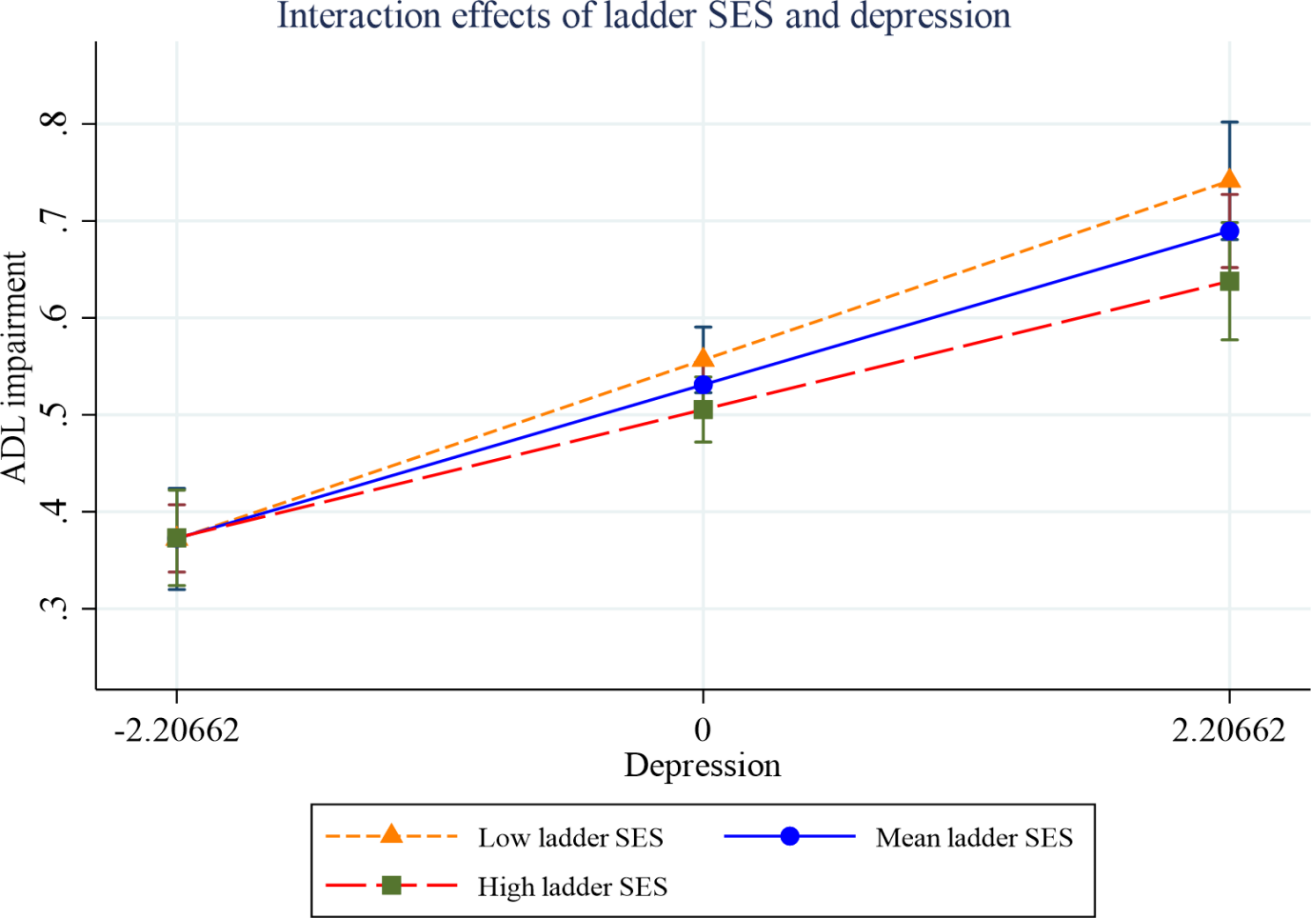
Effect of depression on the relationship between SSS and ADL impairment

**Fig. 3:**
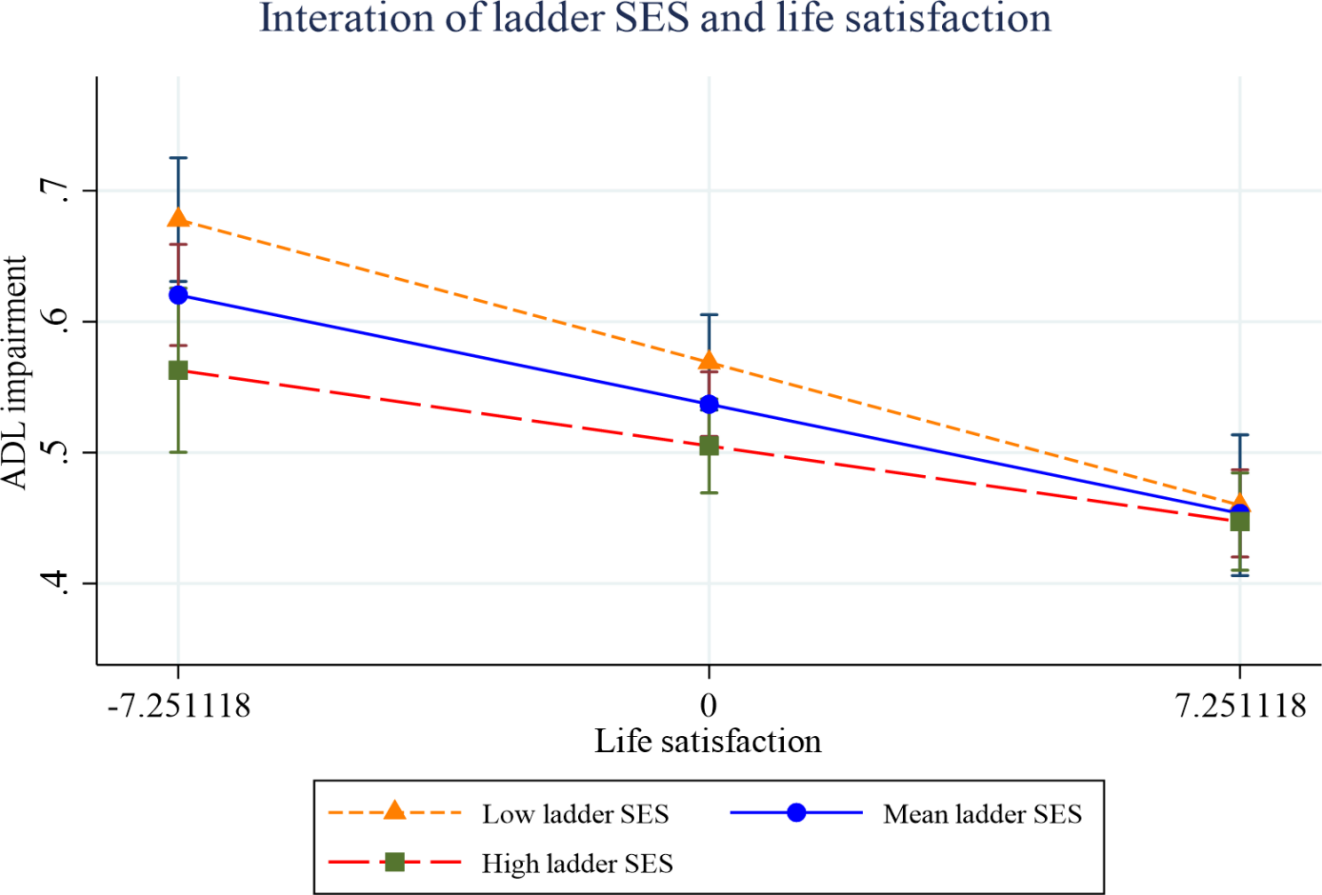
Effect of life satisfaction on the relationship between SSS and ADL impairment

**Fig. 4:**
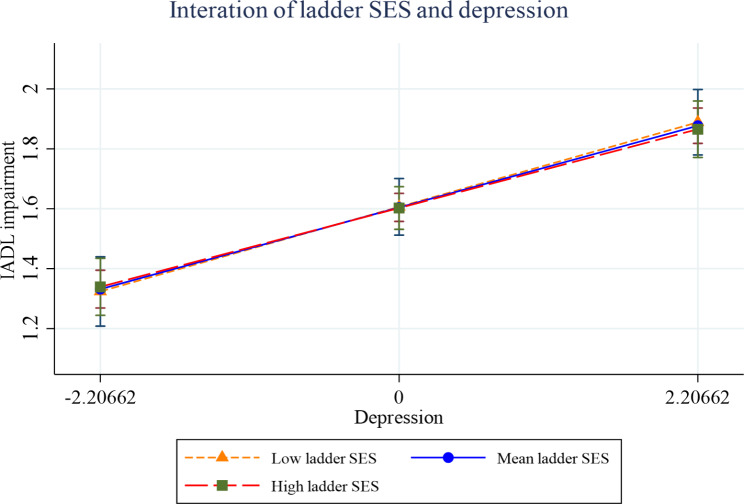
Effect of depression on the relationship between SSS and IADL impairment

**Fig. 5:**
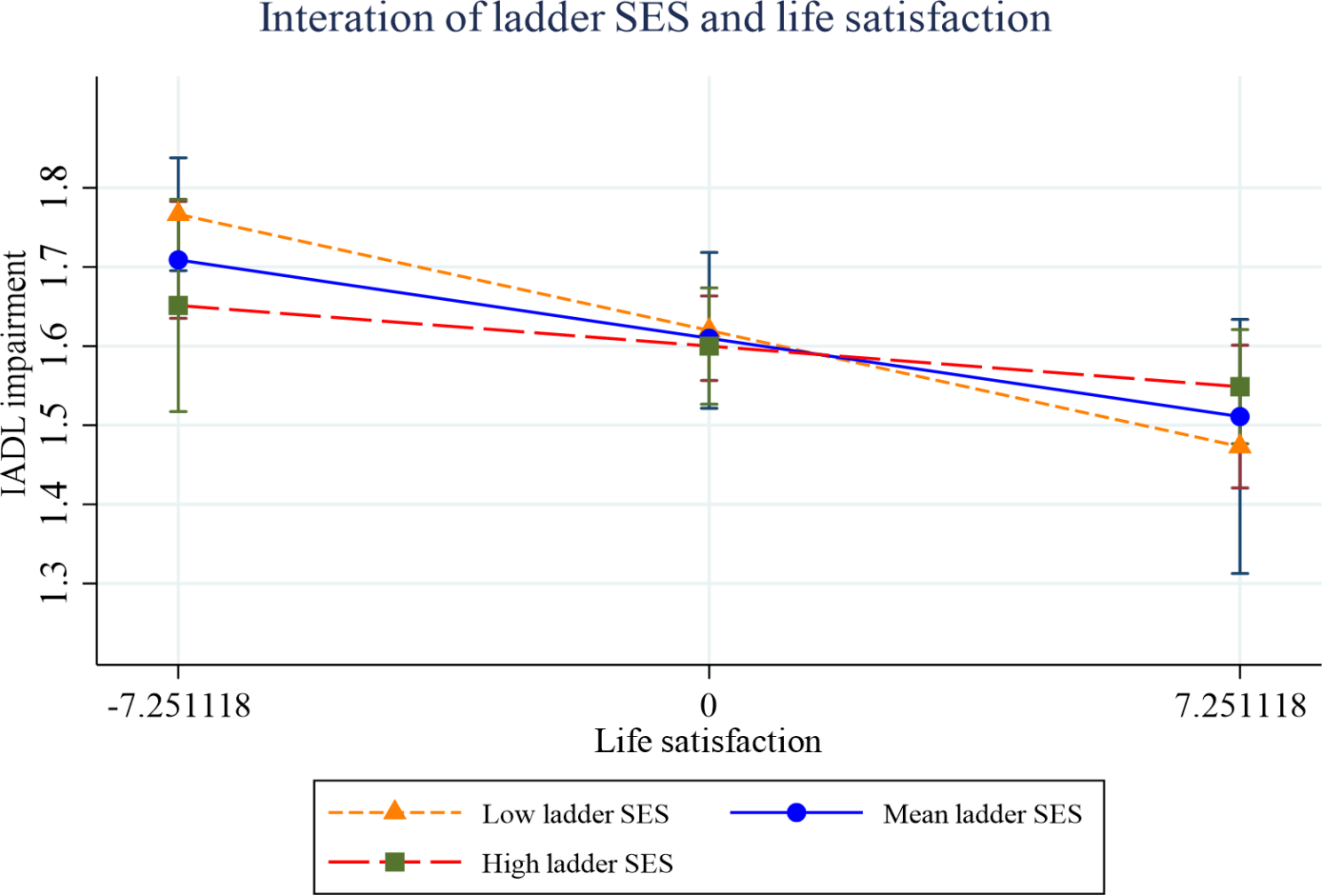
Effect of life satisfaction on the relationship between SSS and IADL impairment

**Fig. 6:**
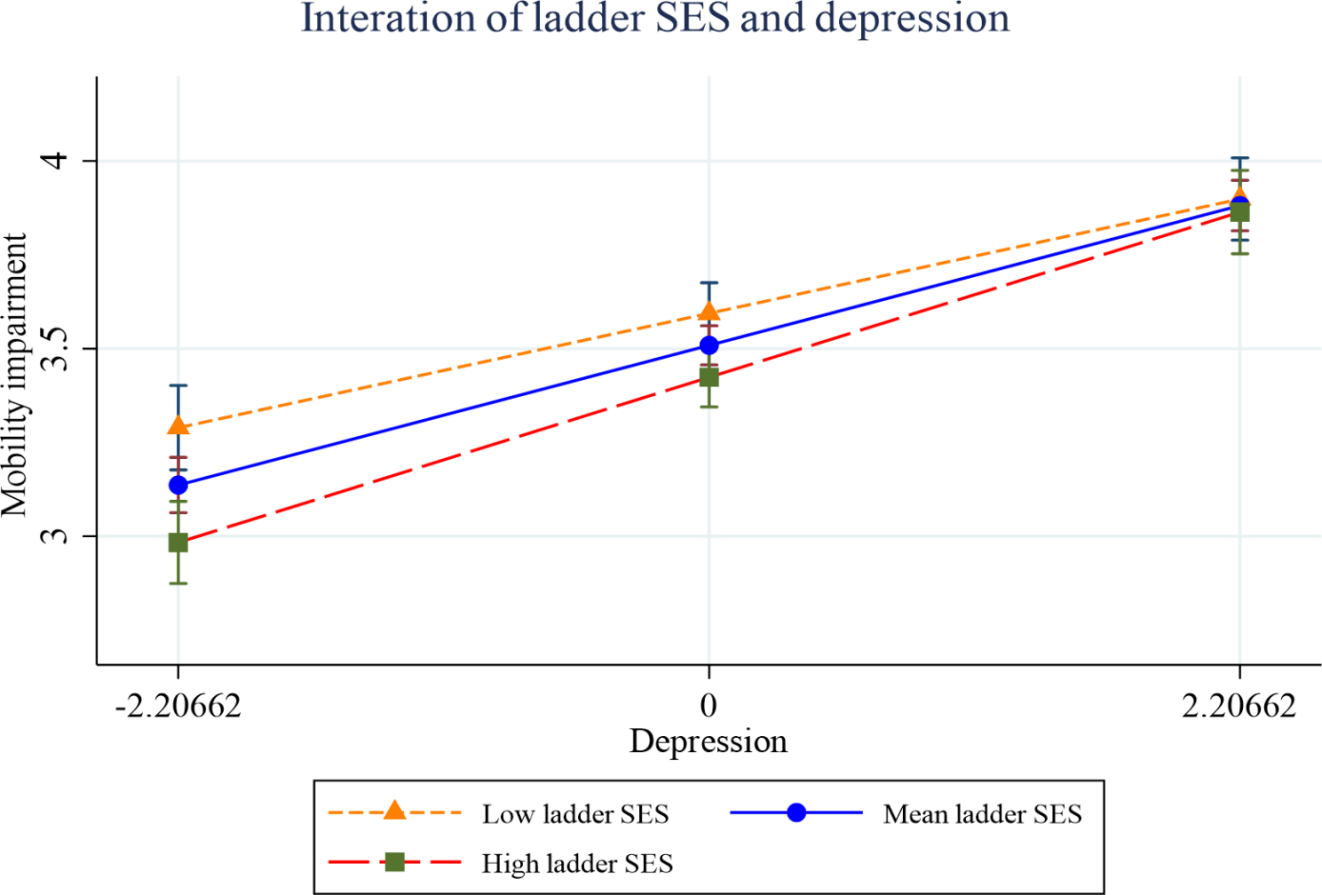
Effect of depression on the relationship between SSS and mobility impairment

**Fig. 7:**
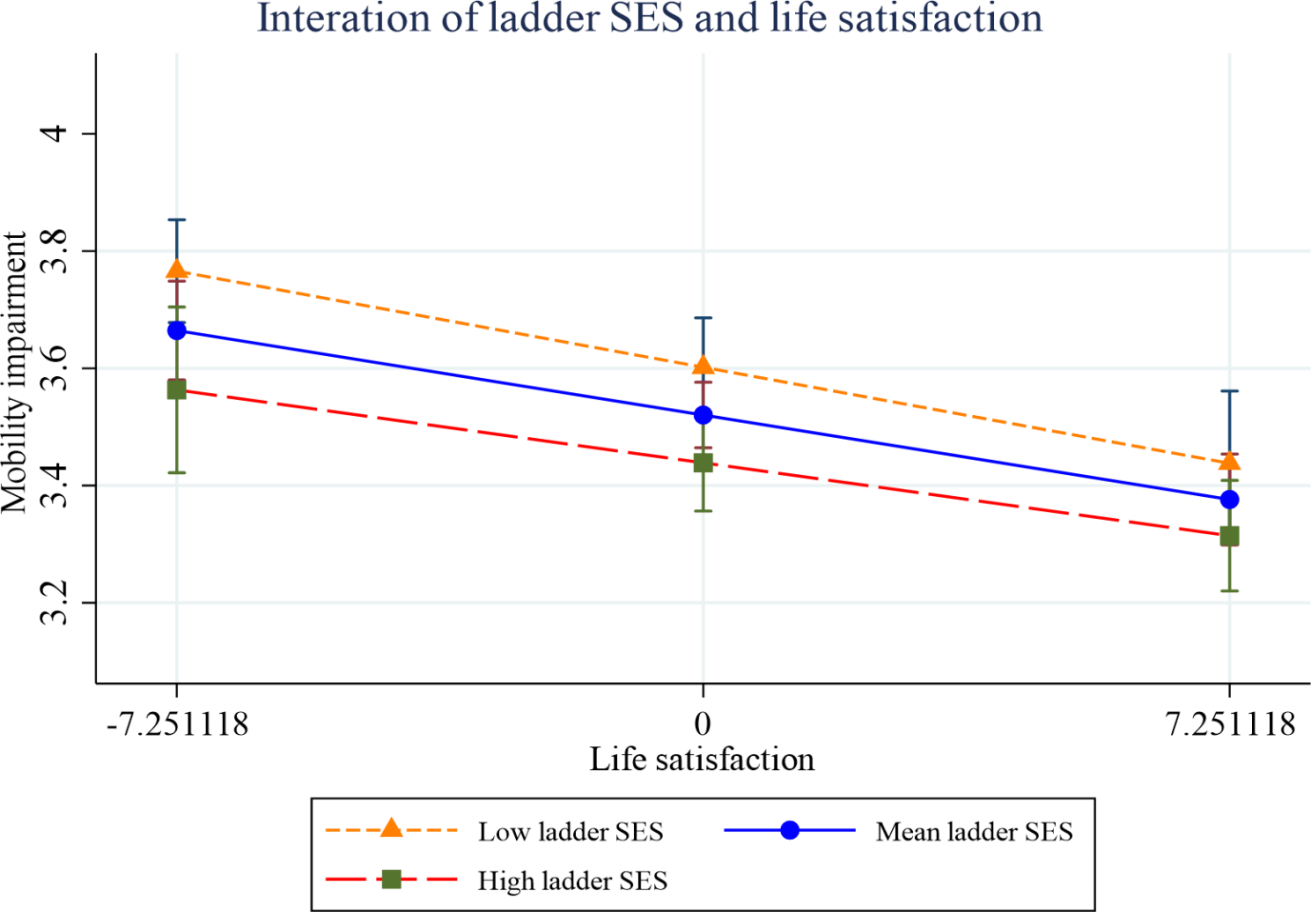
Effect of life satisfaction on the relationship between SSS -mobility impairment

Figures 2, 3, 4, 5, 6 and 7 present the margin plots of the interaction effects of depression and life satisfaction on the associations of SSS with ADL, IADL, and mobility impairments. As can be seen from the plots, depression moderated the association between SSS and mobility impairment (p-value: 0.025). However, it did not moderate the association between SSS and ADL impairment (p-value: 0.137) and SSS and IADL impairment (p-value:0.690). Alternatively, life satisfaction moderated the association between SSS and ADL impairment (p-value: 0.041) and SSS and IADL impairment (p-value: 0.037). However, it did not moderate the association between SSS and mobility impairment (p-value: 0.446).

Table 5 presents the estimates from the mediation analysis. Depression mediated 20.3%, 31.9%, and 18.4% of the associations of SSS with ADL, IADL, and mobility impairments, respectively. Similarly, life satisfaction mediated 23.2%, 52.7%, and 27.2% of the associations of SSS with ADL, IADL, and mobility impairments, respectively, in this study.

**Table 5:** Direct and indirect effects of the SSS-FMIs associations via depression and life satisfaction

	ADL impairment	IADL impairment	Mobility impairment
	aCoef (95% CI)	aCoef (95% CI)	aCoef (95% CI)
SSS			
Total effect	-0.034*** (-0.041 - -0.026)	-0.029*** (-0.040 - -0.017)	-0.084*** (-0.10 - -0.068)
Direct effect	-0.042*** (-0.050 - -0.035)	-0.042*** (-0.054 - -0.030)	-0.10*** (-0.12 - -0.087)
Indirect effect via **depression**	-0.0086*** (-0.0098 - -0.0073)	-0.013*** (-0.015 - -0.011)	-0.019*** (-0.022 - -0.016)
PEM	20.28	31.88	18.39
**SSS**			
Total effect	-0.032*** (-0.040 - -0.025)	-0.020** (-0.032 - -0.0075)	-0.075*** (-0.092 - -0.058)
Direct effect	-0.042*** (-0.050 - -0.035)	-0.042*** (-0.054 - -0.030)	-0.10*** (-0.12 - -0.087)
Indirect effect via **life satisfaction**	-0.0098*** (-0.012 - -0.0080)	-0.022*** (-0.025 - -0.019)	-0.028*** (-0.032 - -0.024)
PEM	23.24	52.69	27.22

*Notes: aCoef: Coefficients adjusted for age, gender, education, marital status, living arrangements, work status, multimorbidity, community involvement, household consumption quintiles, religion and caste, place of residence and regions*

*PEM: Percent of effect mediated*

## Discussion

The present study focused on the association between SSS and FMIs and examined the extent to which this association is mediated and moderated by life satisfaction and depression. Our data support our hypothesis that older Indians who report low SSS also report higher ADL and mobility impairments, and their peers with higher SSS are less likely to report ADL and mobility deficits. SSS remains consequential for measures of functional and mobility outcomes even after accounting for objective SES. This underscores that objective and subjective measures of SES are not interchangeable. Moreover, the association between SSS and FMIs is mediated and moderated by life satisfaction and depression.

That SSS is relevant for FMIs matches findings in past literature on SSS and physical health [27, 35, 41, 42, 44, 45]. Our study contributes to this literature by exploring the linkage between SSS and FMIs among aging adults in India, an otherwise underexamined LMIC, in this body of work. Examining the relevance of SSS for health in different aging populations across different countries is important, given that research points out group-level differences in self-appraised social standing. For example, studies on SSS in the US have suggested that the positive association between low SSS and poor health is less pronounced among Black Americans relative to their non-Hispanic White counterparts [45, 78, 79], and this probably is because individuals from diverse racial, ethnic, and cultural backgrounds may draw on diverse sources for their social status [45, 80, 81]. For example, compared to their non-Hispanic White peers, Black Americans may base their social status on racial identity, group solidarity, and intergenerational harmony [81]. An additional advantage to a study of this type is that examining the health significance of SSS for older adults in a country with different physical, social, family, and financial contexts helps clarify elements of the aging experience that are likely universal and others which may manifest out of broader macrosocial conditions specific to one country or culture, rather than aging in and of itself.

Another way in which our study contributes to the existing literature is that our findings underscore the dual role that life satisfaction and depression play in both mediating and moderating the association between SSS and FMIs. Specifically, the mediating influence of these two factors is evident in our study because low SSS is associated with lower levels of life satisfaction and higher levels of depression —and this, in turn, is found linked to both functional and mobility impairments. Adverse health outcomes like FMIs do not always directly result from low SSS. Rather, the psychosocial stress -- as manifested in reduced life satisfaction and higher levels of depression -- of being socioeconomically disadvantaged is what produces adverse health outcomes [78]. This finding is important because higher life satisfaction and lower depression are associated with greater social engagement, health-promoting behaviors (e.g., regular exercise, healthier nutrition, use of preventive health care), reduced risky behaviors (e.g., smoking, drinking, substance use), and enhanced biologic function (e.g., less hypertension, low inflammation, low levels of cortisol) [50, 52, 82]. Social engagement and better health behaviors, in turn, are critical to sustaining functional health and mobility [82–84].

In contrast to mediation, the moderation analyses reveal that the association between SSS and FMIs differs among older adults based on life satisfaction and depression. Older adults who are more satisfied with their lives experience fewer negative health consequences of SSS. Moreover, those with lower levels of depression may be psychologically better equipped to withstand the otherwise negative repercussions of SSS. Older adults with higher life satisfaction and lower depression may be more likely to appraise their lower social status as challenging rather than threatening, to be optimistic and hopeful about their financial situation, and to make favorable social comparisons. It also is possible that those who are relatively more satisfied and less depressed may increase their engagement in domains where SES may be less important, such as family relationships, friendships, and volunteering.

These findings provide evidence that perceived social standing is a means through which SES is linked to physical health. This finding implies that researchers, providers, and practitioners should redouble efforts to understand how people “feel” relative to others in their social circle instead of merely focusing on the objective markers of SES. Moreover, findings here compel us to consider that if higher SSS is indeed associated with higher life satisfaction and lower depression, then applying specific cognitive-behavioral therapies to minimize a person’s sense of social inferiority may be crucial for sustaining physical health. Most interventions directly tackle health deficits (e.g., increasing access to health care and the use of pharmacologics), and some aim to reduce the stigma attached to lower SES. While such interventions are invaluable, they need to be supplemented by initiatives and efforts to reduce the stress that may accrue due to lower SSS.

### Limitations and future directions

The findings of this study must be considered within the context of important limitations. *First*, as the project is cross-sectional, we cannot make any predictive claims. A more definitive statement about the relationships among SSS, life satisfaction, depression, and FMIs may be reached by using the forthcoming waves of LASI. *Second*, although life satisfaction and depression are consequential in explaining the link between SSS and FMIs, neither fully explains it. Therefore, it would be worthwhile to identify other mediators of this association. For example, SSS may either undermine social relationships or motivate individuals to invest more time and effort into them, and social relationships, and in turn, social support, are powerful predictors of physical health [85]. Stable social relationships and social support may mediate the link between SSS and FMIs; the implications of SSS for FMIs may also be differentially distributed based on the availability, quality, stability, and type of social resources. *Third*, the possibility of reverse causality lingers, given that FMIs, depression, and life satisfaction, each separately and interactively, could negatively shape perceptions of social status. *Fourth*, primary variables of interest, including depression, in our study are self-reported, which may overstate the relationship between SSS and FMIs due to their shared variance. Notwithstanding these limitations, our study is among the first in India to evaluate the impact of both objective SES and SSS on later life FMIs. Moreover, we do so by engaging a sizeable sample of a nationally representative aging population.

## Conclusion

In conclusion, low SSS is associated with FMIs among older Indians. This association persisted even after accounting for objective SES and other conceptually relevant sociodemographic factors. However, the relationship between SSS and FMIs is mediated and moderated by individual characteristics of life satisfaction and depression. This knowledge may give providers and practitioners additional information to identify older adults most susceptible to FMIs.

## Data Availability

The study uses secondary data which is available at the Gateway to Global Aging Data (https://g2aging.org/).

## Declarations

The survey agencies that conducted the field survey for the data collection have collected prior informed consent (signed and oral) for both the interviews and biomarker tests from the eligible respondents in accordance with Human Subjects Protection.

In case of illiterate older people, informed consent was obtained from their legal guardians for the study participation.
